# Plant associated fungal endophytes as a source of natural bioactive compounds

**DOI:** 10.1080/21501203.2020.1870579

**Published:** 2021-01-27

**Authors:** Nilesh Rai, Priyanka Kumari Keshri, Ashish Verma, Swapnil C. Kamble, Pradeep Mishra, Suvakanta Barik, Santosh Kumar Singh, Vibhav Gautam

**Affiliations:** a Centre of Experimental Medicine and Surgery, Institute of Medical Sciences, Banaras Hindu University, Varanasi, India; b Department of Technology, Savitribai Phule Pune University, Ganeshkhind, Pune, India; c Division of Biochemistry, Department of Medical Biochemistry and Biophysics, Karolinska Institute, Stockholm, Sweden; d Chemical Engineering Discipline, Indian Institute of Technology Gandhinagar, Palaj, Gandhinagar, Gujarat, India

**Keywords:** Fungal endophyte, bioactive compound, host endophytes relationship, biological activity

## Abstract

Endophytes are a potent source of bioactive compounds that mimic plant-based metabolites. The relationship of host plant and endophyte is significantly associated with alteration in fungal colonisation and the extraction of endophyte-derived bioactive compounds. Screening of fungal endophytes and their relationship with host plants is essential for the isolation of bioactive compounds. Numerous bioactive compounds with antioxidant, antimicrobial, anticancer, and immunomodulatory properties are known to be derived from fungal endophytes. Bioinformatics tools along with the latest techniques such as metabolomics, next-generation sequencing, and metagenomics multilocus sequence typing can potentially fill the gaps in fungal endophyte research. The current review article focuses on bioactive compounds derived from plant-associated fungal endophytes and their pharmacological importance. We conclude with the challenges and opportunities in the research area of fungal endophytes.

## 1. Introduction

Plant kingdom shows tremendous association with various kinds of microorganisms, which are capable of producing structurally unique and a diverse range of bioactive compounds. These plant-associated microorganisms derived bioactive compounds may act as an antibiotic, inducer, and regulator (AlSheikh et al. 2020; Erb and Kliebenstein 2020; Funes et al. 2020). From the past few decades, various medicinal plants are being utilised for the extraction of natural bioactive compounds, as the semisynthetic and synthetic drugs show long-term negative impact (Subbulakshmi et al. 2012). Natural bioactive compounds are small molecules synthesised by either plants or plant-associated microorganisms such as endophytes. Fungal endophytes reside inside the plant tissues and live symbiotically without showing any apparent harmful symptoms. However, sometimes it is difficult to differentiate the endophytic and pathogenic fungi, especially when both microorganisms show similar kind of genetic signature. Numerous subspecies of fungal endophyte have shown both characteristics, i.e. endophytic as well as pathogenic properties (Schouten 2019a). Fungal endophytes are natural reservoir of novel bioactive compounds with medicinal importance (Newman and Cragg 2016). As per a recent report, more than 70% of the anticancer and antimicrobial agents derived from endophytic fungi are natural bioactive compounds or their derivatives (Newman and Cragg 2020). Some reports have also shown that more than 51% of fungal-endophyte-derived bioactive compounds have unknown structure, which shows the biotechnological significance of such fungal groups to the finding of new drugs (Schulz et al. 2002). The plant kingdom provides shelter to millions of endophytic species, and the species richness and diversity of fungal endophyte depends upon the climatic conditions such as rainfall and atmospheric humidity in which the plant grows (Selvanathan et al. 2011). The chemical profile of the plant could influence the bioactive compounds derived from fungal endophytes (Kusari et al. 2012). Studies have shown that fungal endophytes are potent source of structurally diverse and novel bioactive compounds, belonging to various classes such as flavonoids, alkaloids, steroids, polyphenols, terpenoids, and tannins (Gouda et al. 2016; Rustamova et al. 2019).

In the last decade, biology and chemistry of fungal endophytes associated with plants have emerged as an interesting topic to understand the pharmaceutical importance of bioactive compounds derived from fungal endophytes. A study on relationship between chemical and structural activity provides an insight into the diverse biological activity of bioactive compounds derived from fungal endophytes (Abe et al. 2018). On the other hand, biological studies provide better understanding of fungal diversity, host–endophyte relationship, enhanced production of fungal-endophyte-derived bioactive compounds, and their mechanism of action against various diseases. Bioactive compounds derived from fungal endophytes have shown significant impact in hypocholesterolaemia, immunological diseases, diabetes, and oxidative-stress-related problems, which are briefly described in the later section of the review. Inspite of biological properties, the bioactive compounds derived from fungal endophytes have also been found to be helpful in crop improvement and diminishing the negative effect of abiotic stress through the production of phytohormones such as gibberellins (Khan A. L. et al. 2015). The present review summarises about the plant–endophytes relationship and the possible factor affecting the diversity and distribution pattern of fungal endophytes. In later sections, we have described the fungal-endophytes-derived natural bioactive compounds. Uses of fungal-endophytes-derived bioactive compounds in drug discovery against various diseases and other medical application are also described in brief. The review also presents the challenges and future perspective associated with fungal endophyte research.

## 2. Plant–endophytes relationship

Endophytic fungi colonise inside the host tissue and help in producing the plant hormones, bioactive compounds, and promote the accumulation of secondary metabolites (Shwab and Keller 2008; Waqas et al. 2012). In a symbiotic relationship, plant helps fungal endophyte by providing nutrients, shelter, and seed dissemination, whereas fungal endophytes transform the bioactive compounds synthesised by the host-plant into multifunctional products (Schouten 2019b). Fungal endophytes are also known to influence the biosynthesis of enzymes, phytohormones, and bioactive compounds of plants (Khan Abdul Latif et al. 2016; Satheesan and Sabu 2020). The crosstalk between plant–endophyte and endophyte–endophyte has been reported to trigger the biosynthesis of bioactive compounds (Kusari et al. 2012). However, our understanding of host (plant)–endophyte (fungal) relationships is still inadequate in terms of biochemistry and physiology. The intricacies of the fungal endophytes and host plants are thought to be varying from microbe to microbe and host to host (Verma et al. 2009). Endophytes are known to be harboured inside the living plant host that may be growing in extremes of weather conditions including deserts, geothermal soils, and coastal regions (Ali et al. 2018). Therefore, revealing the relationships of the host plant and fungal endophytes may aid in the manufacturing of drugs with enhanced quality through the application of modern biological tools and manipulation of medicinal plant growth conditions (Firáková et al. 2007). The genomic integrity of fungal endophytes could be modified through the application of the latest biological tools such as clustered regulatory interspaced short palindromic repeats-Cas9 (CRISPR-Cas9), zinc finger nuclease (ZFN), and transcription activator-like effector nucleases (TALEN) (Chen and Gao 2013; El-Sayed et al. 2017; Li D et al. 2017). The host–endophyte and endophyte–endophyte relationship could be explored in a better way through genetic engineering by CRISPR-Cas9 techniques along with the advancement of specific fungal endophytic strain. Application of CRISPR-Cas9 to fungal endophytes can help to obtain an enhanced quantity of specific bioactive compounds (El-Sayed et al. 2017; Yan et al. 2018). Nielsen et al. applied the CRISPR-Cas9 technique to identify a gene that produces polyketide-nonribosomal peptide from *Talaromyces atroroseus* (Nielsen et al. 2017). In similar ways, modern biological tools are also competent for exploration of the relationship of host-plant, and subsequently, the bioactive compounds can be efficiently produced for drug development.

An understanding of potential factors affecting the growing condition of the host plant is required which ultimately affects the fungal colonisation and the production of bioactive compounds. For example, a particular factor can influence the distribution ranges of host plants which in turn influence the species of fungal endophytes, their richness, spore germination, and metabolism. Furthermore, the production and extraction of bioactive compounds from endophytic fungi are affected by various factors including environmental condition, geographical location, season of sample collection, and genotype of host and endophyte (Shukla et al. 2014; Morales-Sánchez et al. 2020). Besides this, some of the other limiting factors including gene overexpression, co-culturing, precursor feeding, and use of adsorbent resins are also known to modulate the production of bioactive compounds (Wang M et al. 2016; Li H et al. 2018). Some of the factors known to affect the production of bioactive compounds are described below.

### 2.1 Atmospheric moisture and temperature

The population of fungal endophytes in plant tissues can be affected indirectly by environmental conditions such as humidity, temperature, and illumination. It is reported that fungal endophytes associated with medicinal plants could synthesise a high amount of nutrients under high mean annual atmospheric moisture and low mean annual sunshine hour (Wu L et al. 2013). For example, in the Yellowstone National Park, fungal endophyte *Curvularia protuberata* associated with grass *Dichanthelium lanuginosum* has been reported to survive at high temperature (Márquez et al. 2007). Similarly, Loayza et al. have evaluated the functional aspects of fungal endophyte *Diplodia mutila* screened from *Iriartea deltoidea*. Under the influence of high illumination (408 ± 17.3 μmol mˉ2sˉ^1^ ± SE), *Diplodia mutila* are shown to cause tissue necrosis and cell death through inducing the ROS (reactive oxygen species) production. On the contrary, low illumination (208.2 ± 6.1 μmol mˉ2sˉ^1^ ± SE) maintains endosymbiotic development (Alvarez-Loayza et al. 2011). Recently, it has been reported that under high temperature, quantity of bioactive compounds achieved from carrot peel was maximum with excellent anti-oxidant activity (Nguyen and Le 2018). However, only a few and specific fungal endophytes could survive successfully under unfavourable conditions such as cold climate, temperate condition, insignificant concentration of oxygen, respiration rates, and pH. Therefore, colonisation of a limited number of fungal species is found in the respective host plant (Shu et al. 2010).

### 2.2 The geographical location of host plants

The majority of the fungal endophytic community, their diversity, and abundance are shown to be influenced by the geographical location of the host plant and local environment (Mo et al. 2008). Statistical analysis of fungal communities of white snakeroot (*Ageratina altissima*) demonstrated that a community of fungal endophytes strongly resists the anthropogenic and biotic disturbance (Christian et al. 2016). It has been shown that fragmentation of the environment can produce negative impact on the occurrence of endophytic fungi. Decreased fragment size has decreased the colonisation of fungal endophyte (Helander et al. 2007). Based on geographical location, a varying degree of the fungal endophytes are isolated and identified from the plants of different zones including Antarctic (Yu et al. 2014), Arctic (Botnen 2020), Temperate zone (Unterseher 2011), and Tropical zone (Ting 2020). Plants growing in the tropical zone are reported to produce an increased amount of fungal-endophyte-derived bioactive compounds in comparison to any other zonal plant-associated endophytes (An 2002; Arnold and Lutzoni 2007).

### 2.3 Age of host plant tissue

Wide range of fungal endophyte species colonises in plant tissues such as vascular ducts, parenchyma, and dermis of different age groups. Study on *Calotropis procera* shows that species richness and colonisation of fungal endophytes were highest in aged leaves. The study also showed the presence of fungal endophytes of *Xylaria* sp. in young leaves (Nascimento et al. 2015). Previous studies have suggested that colonisation of fungal endophytes depends on season and type of tissue (i.e. stem, root, leaf, stem bark, twig) (Tejesvi et al. 2005; Mishra et al. 2012). Thus, age and type of host plant tissue assist the colonisation and interaction of fungal endophytes to their host plants.

### 2.4 Genetic background

The relationship of fungal endophyte with their host plants (i.e. either mutualistic or parasitic) depends upon the host genotype, genotype of the endophyte, and environment in which they grow (Unterseher and Schnittler 2010; Salam et al. 2017). Relationship between fungal endophyte and host plant is established by minor variations in gene expression of fungal endophyte against the reaction of host plant or through identification of host plant and fungal reaction (Estrada et al. 2013; Sarsaiya et al. 2019). Therefore, symbiotic association, i.e. positive, negative, or neutral, is managed by minor variation in genetic constituent of both fungal endophyte and host plant. For example, signal molecule Nod factor Lipo-chitooligosaccharides (LCO) activates the common symbiotic pathway in arbuscular mycorrhizal and rhizobia-legume associations (Gough and Cullimore 2011). A study has revealed that colonisation of *Mucor* sp. in *Arabidopsis thaliana* could be promoted by the secretion of strigolactone (SL) from host plant root (Rozpądek et al. 2018). Therefore, it can be concluded that the genetic background of host plant influences the colonisation of fungal endophyte, which in turn affects the qualitative and quantitative extraction of fungal-endophytes-derived bioactive compounds.

## 3. Endophytes: reservoir of bioactive compounds

**Figure 1.**
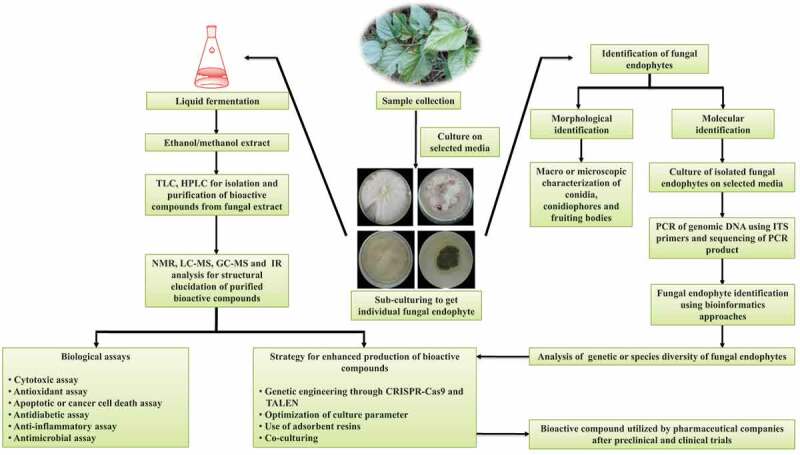
**Sequential events involved in the isolation and characterisation of bioactive compounds derived from fungal endophytes**. For the isolation of a fungal endophyte associated with a medicinal plant, plant tissue samples are grown on selected media. After a certain period of a time, fungal endophytes grow on a media plate. Further, individual fungal strain can be identified using microscopic and molecular approaches after sub-culturing of fungal endophytes. Molecular identification involves isolation of genomic DNA of fungal endophytes followed by polymerase chain reaction (PCR) of internal transcribed spacer (ITS) region. BLASTn analysis of raw sequences obtained from sequencing of PCR products leads to the identification of the fungal strain. For large-scale production of the fungal strain, strains are grown in liquid culture. Fungal-endophyte-derived bioactive compounds are isolated in ethanol or methanol extract. Biological assays are applied to test the efficiency of extracted bioactive compounds. Isolated bioactive compounds are subjected to nuclear magnetic resonance (NMR), gas chromatography-mass spectrometry (GC-MS)-based studies for the molecular identification and quantification. Genome editing approach may be applied to enhance the production of most effective bioactive compounds which could be further tested for drug discovery and development after preclinical and clinical trials

Fungal endophytes are potent source of a wide range of bioactive compounds. Bioactive compounds derived from fungal endophytes are utilised for drug discovery to manage various health ailments. The entire procedure involved in the isolation and characterisation of bioactive compounds from fungal endophytes to eventually commercialise them is illustrated in **Figure 1**. Few notable bioactive compounds such as paclitaxel, podophyllotoxin, vinca alkaloids, camptothecin, hypericin, emodin, azadirachtin, and deoxypodophyllotoxin are isolated from plant-associated fungal endophytes (Kaul et al. 2012). Some of the well-known fungal genera such as *Penicillium, Fusarium, Aspergillus, Sclerotium, Myxormia, Alternaria, Colletotrichum, Cladosporium, Diaporthe,* and *Curvularia* are excellent source for the production of bioactive compounds (Chepkirui and Stadler 2017; Toghueo 2020). Along with the aforementioned fungal genera, several other endophytes are also known to produce bioactive compounds with a significant role in disease management (Jia et al. 2016). Fungal-endophytes-derived bioactive compounds are classified under broad functional groups – alkaloids, steroids, flavonoids, phenolic acids, benzopyranones, quinines, tannins, xanthones, terpenoids, and many others. Bioactive compounds isolated from the extract of fungal endophytes can be purified and characterised through high-performance liquid chromatography (HPLC), thin-layer chromatography (TLC), nuclear magnetic resonance (NMR), infrared spectroscopy (IR), matrix-assisted laser desorption/ionisation – time of flight (MALDI-TOF), electron spray ionisation (ESI), fast atom bombardment (FAB). Some of the major bioactive compounds utilised in the management of human health ailments are discussed below.

### 3.1 Paclitaxel

**Figure 2.**
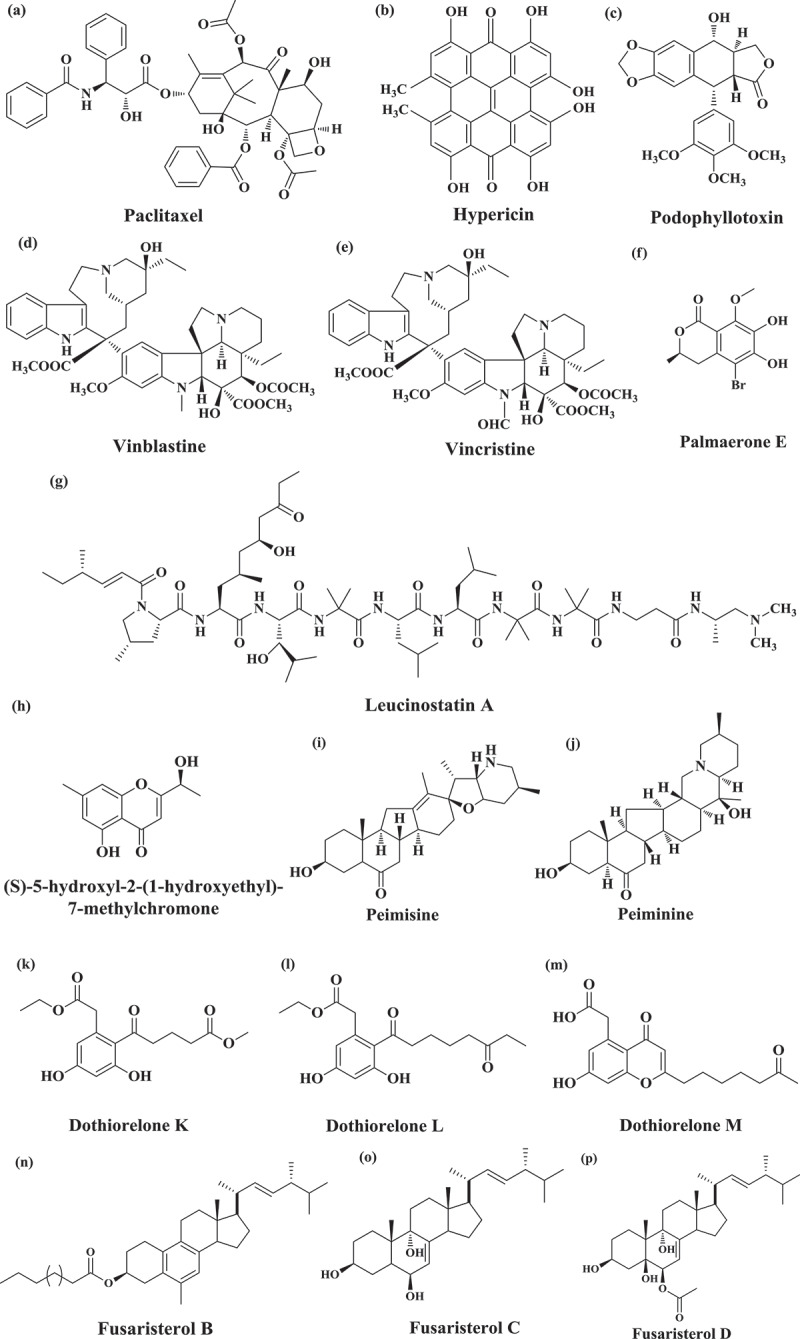
**Chemical structure of bioactive compounds derived from the fungal endophytes. (a)** Paclitaxel, **(b)** Hypericin, **(c)** Podophyllotoxin, **(d)** Vinblastine, **(e)** Vincristine, **(f)** Palmaerone E ((R)-5-bromo-6,7-dihydroxy-8-methoxy-mellein), **(g)** Leucinostatin A, **(h)** (S)-5-hydroxyl-2-(1-hydroxyethyl)-7-methylchromone, **(i)** Peimisine, **(j)** Peiminine, **(k)** Dothiorelone K, **(l)** Dothiorelone L, **(m)** Dothiorelone M, **(n)** Fusaristerol B, **(o)** Fusaristerol C, **(p)** Fusaristerol D

Paclitaxel (*C*_47_*H*_51_*NO*_14_) (**Figure 2a**) is an anticancer (“cytotoxic” and “antineoplastic”) drug used in the chemotherapeutic treatment of numerous cancers including lung, breast, ovarian, prostate, bladder, and melanoma. The generic name of paclitaxel is taxol. Taxol belongs to class “taxane” and is a polycyclic diterpenoid. Initially, the presence of taxol was determined in the inner bark of *Taxus brevifolia* (Pacific yew tree) in 1971 (Wani et al. 1971). Taxol production is not limited to particular fungus species (Gupta S et al. 2019). Numerous fungal endophytes are known to colonise in different host plant including angiosperms that produce taxol such as *Seimatoantlerium nepalense* from *Taxus wallichiana* (Bashyal 1999), *Seimatoantlerium tepuiense* from *Venezuelan Guyana* (Strobel Gary A, Ford Eugene, et al. 1999); *Metarhizium anisopliae, Pestalotiopsis terminaliae,* and *Tubercularia* sp. fungal strain TF5 in batch culture (Gangadevi and Muthumary 2009; Hussain et al. 2015). Moreover, a list of other fungal endophytes including *Fusarium, Mucor, Alternaria, Cladosporium, Phoma, Botryodiplodia, Metarhizium, Periconia, Pestalotiopsis, Botrytis, Taxomyces, Aspergillus, Tubercularia, Pestalotia*, and *Pithomyces* are also reported to produce paclitaxel or its analogues (Zhou et al. 2010; Kasaei et al. 2017).

Initially, the fungal crude extract was shown to possess an excellent cytotoxic activity on the cancer cell lines (Belotti et al. 1996). Later on, paclitaxel entered towards different phases of clinical trials after its potency as an anticancer bioactive compound was proved. However, the foremost hurdle was faced in terms of the low quantity of taxol production. After years of research, the efforts came out with the identification and isolation of taxol precursor, i.e. deacetylbaccatin III from European yew tree *T. baccata* (Hook et al. 1999; Truus et al. 2012). Recently, genome sequence analysis of endophyte *Penicillium aurantiogriseum* revealed how anticancer compound paclitaxel is produced (Yang Y et al. 2014). Identification of fungal endophytes having the ability to produce taxol is now possible through polymerase chain reaction (PCR) based methods. Heinig et al. screened the fungal endophyte *Taxomyces andreanae* associated with the inner bark of *Taxus* spp. plant to re-examine whether endophytic fungi could produce taxol independently or not. The genome sequence analysis of fungal endophyte *Taxomyces andreanae* revealed an interesting fact that taxol could not be synthesised independently from endophytes (Heinig et al. 2013).

### 3.2 Hypericin

4,5,7,4ʹ,5ʹ,7ʹ-Hexahydroxy-2,2ʹ-dimethylnaphthodianthrone (Hypericin) (**Figure 2b**) is a naturally occurring naphthodianthrone compound, the main constituent of *Hypericum* plant (St. John’s Wort) (Kusari et al. 2008). The stem tissue of the medicinal plant *Hypericum perforatum* was screened with the aim of isolation of fungal endophytes. An anthraquinone derivative emodin has ability to synthesise hypericin. It is one of the top-selling natural herbal medications because of its antioxidant, antiviral, antibiotic, and non-specific kinase inhibition properties. Moreover, hypericin is widely being applied as an antidepressant, anti-inflammatory, injury healer, antimicrobial, seasonal affective disorder, as well as sinusitis reliever (Strobel 2003; Zhao et al. 2010). Currently, the role of hypericin in the treatment of cutaneous T-cell Lymphoma is under investigation. In addition to *Hypericum* sp., other species are also reported to produce hypericin such as the basidiomycete genus *Dermocybe* (Garnica et al. 2003). The gene Hyp-1 is also shown to be involved in the final step of biosynthetic pathway of hypericin (Bais et al. 2003). Kusari et al. screened *H. Perforatum* and showed that fungal endophyte *Thielavia subthermophila* produces hypericin. They have also suggested that production of hypericin is found even in the absence of candidate gene Hyp-1 (Kusari et al. 2009). Later on, leaves from *H. perforatum* were harvested for the screening of fungal endophytes. Fungal strain *Aspergillus* sp. TJ23 was isolated and identified as a producer of asperetide, a polyketide and asperanthone, a derivative of prenylxanthone (Qiao et al. 2018).

### 3.3 Podophyllotoxin

Podophyllotoxin (**Figure 2c**) is an anticancer drug employed as a precursor in the biosynthesis of cytotoxic bioactive compounds including etopophose phosphate, teniposide, and etoposide (Ardalani et al. 2017). Teniposide and etoposide are being utilised in various cancer such as lung cancer, testicular cancer, and other types of leukaemia’s and solid tumours (Gupta RS et al. 1987; Kiran et al. 2018). Podophyllotoxin is broadly distributed among plant genera including *Podophyllum, Dysosma, Diphylleia,* and *Juniperus* (Li J et al. 2013). It binds irreversibly to tubulin for inhibition of mitotic cell division and interferes with the dynamic equilibrium inducing arrest of the cell cycle at the G2/M phase (Zhang X et al. 2018). In one of the study, podophyllotoxin (yield of 277 μg/g dry weight mycelia) has been extracted using high-performance liquid chromatography (HPLC) and ultra-performance liquid chromatography-quadrupole-time of flight mass spectrometry analyses (UPLC–QTOF MS) produced from a fungal endophyte of *Fusarium* strain WB5121 (Tan et al. 2018).

### 3.4 Vinca alkaloids

Vinca alkaloids belong to the terpenoid group and are derived from vindoline and catharanthine monomer (Noble 2016). Vinca alkaloids isolated from *Catharanthus roseus* (vernacular name “periwinkle”) were initially used as oral antidiabetic medicine before the discovery of insulin (Strobel and Hess 1997). However, experimental analyses failed to generate any positive result of antidiabetic property (Johnson et al. 1963; Strobel and Hess 1997; Noble 2016). Vinca alkaloids are found to be effective in cancer such as nephroblastoma and acute lymphoblastic leukaemia as it reduces leukocytes (Moudi et al. 2013). Other alkaloids with anticancer potency are vinblastine, vinleunosine, vinrosidine, and vincristine (Moudi et al. 2013; Noble 2016). Vinblastine (**Figure 2d**) and vincristine (**Figure 2e**) are the terpenoid indole alkaloids isolated from *Catharanthus roseus* that inhibit cell cycle at M phase and irreversibly binds with microtubule and spindle protein in S phase. It is used in the treatment of cervical cancer and Hodgkin lymphoma (Kaur R et al. 2014). From the fungal endophyte *Curvularia verruculosa* associated with the leaves of *C. roseus,* vinblastine compound was isolated which showed cytotoxic effect against HeLa cell line with IC_50_ 8.5 µg/mL (Parthasarathy et al. 2019).

### 3.5 Palmaerones

Seven new bioactive compounds, palmaerones A-G (2 chlorinated and 5 brominated bioactive compounds) were derived from fungal endophyte *Lachnum palmae* associated with *Przewalskia tangutica*. These bioactive compounds were analysed for antimicrobial activity against three fungal species (*Candida albicans, Cryptococcus neoformans*, and *Penicillium* sp.,) and two bacterial species (*Staphylococcus aureus* and *Bacillus subtilis*) (Zhao M et al. 2018). Out of seven, only palmaerone E ((R)-5-bromo-6,7-dihydroxy-8-methoxy-mellein) (**Figure 2f**) showed significant antimicrobial activity against all the microbial strains with minimum inhibitory concentration value in the range of 10–55 mg/ml. The cytotoxicity analysis of these bioactive compounds was performed against the three human cancer cell lines: SGC7901, HepG2, and HL-60. Palmaerone E showed weak cytotoxicity against HepG2 cells with inhibitory concentration value of 42.8 mM.

### 3.6 Leucinostatin A

Leucinostatin A (*C*_62_*H*_111_*N*_11_*O*_13_) (**Figure 2g**) is a nonapeptide complex which shows diverse range of biological activity against numerous cancer cell lines (Li H et al. 2005). Leucinostatin A has been reported to reduce the proliferation of prostate cancer cell growth. Leucinostatin A targets insulin-like growth factor-I (IGF-1) of prostate stromal cells and thereby show the anticancer property (Kawada et al. 2010). Leucinostatin A also shows phytotoxic, antiviral, antimalarial, and antifungal activities (Deshmukh et al. 2018). Leucinostatin A is derived from a fungal endophyte of *Acremonium* sp. in liquid culture, isolated from *T. baccata. Acremonium* sp. is also known to produce a glycosylated bioactive compound leucinostatin A β-di-O-glucoside, which is analogue of leucinostatin A (Strobel and Hess 1997). The bioactive compound leucinostatin A β-di-O-glucoside show cytotoxic activity against BT-20 (breast cancer cell line) with a value of LD_50_ >25 nM, while leucinostatin A had LD_50_ value of 2 nM against the same cell line. Genome mining studies for *Purpureocillium lilacinum* deciphered a cluster of about 20 genes which are responsible for the biosynthesis of leucinostatin A and B (Wang G et al. 2016).

### 3.7 Other bioactive compounds

A new bioactive metabolite (S)-5-hydroxyl-2-(1-hydroxyethyl)-7-methylchromone was derived from fungal endophyte *Bipolaris eleusines*, associated with fresh potato (**Figure 2h**). This compound showed weak antibacterial activity against *Staphylococcus aureus* subsp. *aureus*, and the rate of inhibition was 56.3% at 128 mg/ml of concentration (He et al. 2019). *Fusarium* sp. associated with the plants of *Fritillaria* sp. has the ability to produce steroidal alkaloids such as peimisine (**Figure 2i**) and peiminine (**Figure 2j**) (Pan et al. 2014). Peiminine show anticancer property by inhibiting the proliferation and colony formation of cells. It might be involved in cell cycle arrest and autophagic flux (Pan et al. 2014). Peiminine changes the expression of microtubule-associated protein 1A/1B-light chain 3 (LC3), p62, and the network of cyclin-dependent kinase (cyclin D1/CDK) by downregulating the expression of phosphorylative derivatives Akt, glycogen synthase kinase 3 beta (GSK3B), 5ʹ AMP-activated protein kinase (AMPK), and Unc-51 Like Autophagy Activating Kinase 1 (ULK1) (Zhao B et al. 2018).

Three bioactive compounds, dothiorelone K (**Figure 2k**), dothiorelone L (**Figure 2l**), and dothiorelone M (**Figure 2m**), were derived from fungal endophyte *Dothiorella* sp. ML002 associated with the stem tissue of mangrove plant *Xylocarpus granatum* (Zheng et al. 2019). These bioactive compounds were cytosporone derivatives. The anti-diabetic roles of these compounds were evaluated by testing the α-glucosidase inhibitory activity. The bioactive compound dothiorelones K and L displayed inhibitory activities with the IC_50_ value of 22.0 and 77.9 µg/ml, respectively. From the fungal endophyte *Fusarium* sp. associated with the root tissue of plant *Mentha longifolia,* three ergosterol derivatives (Fusaristerol B (**Figure 2n**), Fusaristerol C (**Figure 2o**) and Fusaristerol D (**Figure 2p**)) along with two known compounds ((22E,24 R)-ergosta-7,22-diene-3b-ol and (22E,24 R)-5b,8b-epidioxyergosta-22en-3b-yl decanoate) were isolated. Fusaristerol B, Fusaristerol C, and Fusaristerol D possessed 5-lipoxygenase (5-LOX) inhibitory potential with IC_50_ value of 3.61 µM, 7.01 µM, and 4.79 µM, respectively (Khayat, Ibrahim et al. 2019). Thus, the derivatives of ergosterol could be potential bioactive compounds in the formulation of anti-inflammatory drugs.

## 4. Biological properties of bioactive compounds

**Figure 3.**
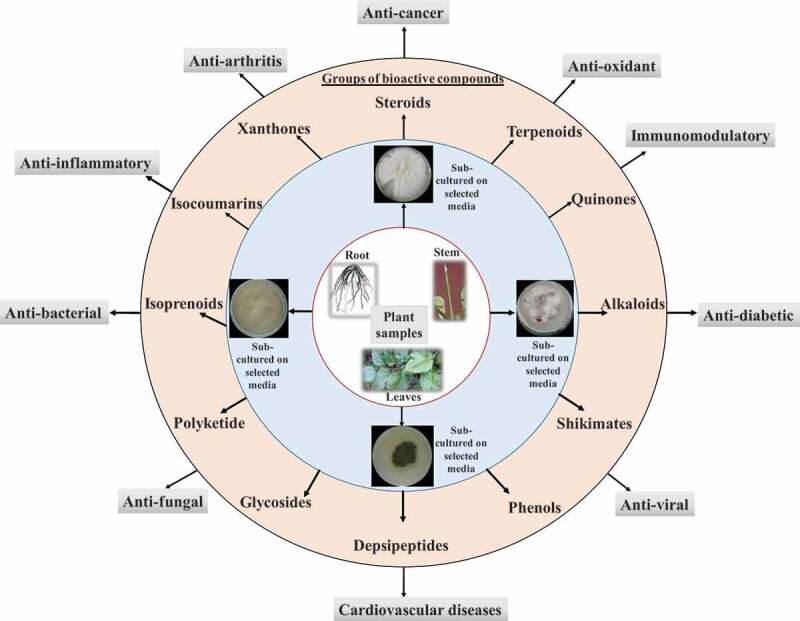
**Bioactive compounds derived from fungal endophytes with pharmacological relevance**. Fungal endophytes harbour inside almost every plant tissue. Fungal endophytes can be isolated from stem, root, and leaves by culturing in the selected growth media. Fungal endophytes are potent source of number of bioactive compounds which can be utilised in the treatment of number of human health ailments

Fungal endophytes are excellent source of a wide range of bioactive compounds with pharmacological importance (**Figure 3**). Few pharmacologically important bioactive compounds derived from fungal endophytes are discussed below.

### 4.1 Fungal-endophyte-derived anticancer compounds

Cancer is the second leading cause of death globally. Due to the high mortality rate associated with cancer, search for novel anticancer drugs is in progress. In the last two decades, fungal endophytes have achieved much attraction of scientific community due to their immense potential to synthesise various types of anticancer compounds including Taxol, Podophyllotoxin, Vinca alkaloids, Graphislactone A, Rohitukin, Cytochalasin 1–3, Fusarithioamide A, and Malformin E. The anticancer compounds derived from endophytic fungi belong to various classes including Alkaloids, Polyketides, Ergochromes, Depsipeptides, Chromones, Benzo[*j*]fluoranthenes, Aldehydes, Quinones, Depsidones, Esters, Lignans, Cyclohexanones, Xanthones, Sesquiterpenes, and Diterpenes (Uzma et al. 2018; Li S-J et al. 2018). The bioactive compound derived from endophyte *Aspergillus iizukae*, associated with the plant *Silybum marianum* is silybin A and silybin B. Silybin A and B are known to have an anti-inflammatory and antitumor activity (El-Elimat et al. 2014; Surai 2015). Bioactive compounds derived from fungal endophytes are reported to be a precursor in the synthesis of many anticancer compounds; for example, the anticancer compound camptothecin is an important precursor of irinotecan and topotecan having anticancer activity. Camptothecin and its analogues are under clinical trials against solid tumours of lung, liver, and ovary and are found to be very effective (Choi et al. 2011). Camptothecin is not water-soluble but its water-soluble derivatives such as Camptosar and Hycamtine are used against colorectal carcinomas and ovarian cancer, respectively (Li F et al. 2017). Some of the major anticancer bioactive compounds derived from fungal endophytes are mentioned in **Table 1**.

**Table 1.** Anticancer activity as conferred by bioactive compounds isolated from fungal endophytes associated with host plants

Host plants	Fungal endophytes	Bioactive compounds	Mode of action	References
*Salacia oblonga*	*Alternaria* spp., *Fusarium solani, Aspergillus niger*	Taxol	Disruption of microtubule equilibrium.	(Roopa et al. 2015)
*Podocarpus gracilior pilger*	*Aspergillus terreus*	Taxol	Trigger tubulin polymerisation through binding with tubulin β-subunits heterodimer and, disrupt tumour cells division	(Stahlhut et al. 1999)
*Camptotheca acuminata*	*Fusarium solani*	Camptothecin	Topoisomerase-I inhibition.	(Ran et al. 2017)
*Catharanthus roseus*	*Fusarium oxysporum*	Vinblastine and Vincristine	Microtubule destabilising agent, cell cycle inhibition at metaphase of mitosis.	(Kumar et al. 2013)
*Podophyllum* sp., *Juniperus recurva*	*Fusarium oxysporum, Trametes hirsuta*	Podophyllotoxin glycoside, 4 -demethylpodophyllotoxin,	Irreversibly binds to tubulin and inhibit mitotic division thus, inducing arrest in G2/M phase of cell cycle.	(Kour et al. 2008)
*Solanum nigrum* L.	SNFSt, SNFL and SNFF	Solamargine	Arrest cell in G2/M phase, interferes with the function and structure of cancer cell membrane, blocks the anti-apoptotic pathway of NF-kβ.	(Atanu et al. 2011, El‐Hawary et al. 2016)
*Piper nigrum*	*Colletotrichum gloeosporioides*	Piperine	G2/M phase arrest in cancer cells.	(Chithra et al. 2014)
*Capsicum annuum*	*Alternaria alternata*	Capsaicin	Induced apoptosis in HL-60 cells.	(Devari et al. 2014)
*Silybum marianum*	*Aspergillus iizukae*	Flavonolignans, silybin A, silybin B and isosilybin A	Promote tubulin polymerisation inhibiting cell division	(El-Elimat, Raja, Graf, Faeth, Cech and Oberlies 2014)
*Panax ginseng*	*Paecilomyces* sp	Ginsengnosides-Rg3, Rh2	Inhibit the proliferation of T24 cells	(Zhang et al. 2006)
*Tabebuia rosea*	*Aspergillus TRL1*	Pulchranin A	Inhibition of cyclin-dependent kinases such as CDK1, CDK2 and CDK4	(Moussa et al. 2019)
*Sinopodophyllum* *hexandrum*	*Pestalotiopsis adusta*	Pestalustaine B	Induce apoptosis	(Xiao et al. 2018)
*Hypericum* *perforatum*	*Emericella* sp. TJ29	Emeridones A–F	–	(Li et al. 2019)
*Pogostemon cablin*	*Cerrena* sp. A593	Triquinane-type sesquiterpenoids Cerrenins D and E	–	(Liu et al. 2018)

### 4.2 Bioactive compounds derived from fungal endophytes for treating immunological disorders

Immunosuppressive drugs are routinely used to treat auto-immune diseases such as Crohn’s disease, rheumatoid arthritis, psoriasis as well as for the prevention of allograft rejection. Chemical drugs like Mycophenolate mofetil and Cyclosporine have many side effects (like hyperglycaemia, osteoporosis, nausea, vomiting, loss of appetite, and diarrhoea) (Yang CW et al. 2003; Fotiadis et al. 2005). To overcome such problems, there is a call for the development of better immunosuppressive agents. Therefore, fungal endophytes are being studied as an alternative source for the compounds to treat immunological disorders. Screening of fungal endophyte *Fusarium subglutinans* results in isolation of Subglutinols A and B having immunosuppressive ability (Lee et al. 1995). In one of the recent reports, a dibenzofurane compound mycousine was produced by fungal endophyte *Mycosphaerella nawae* ZJLQ129, associated with the leaf of plant *Smilax china* (family *Liliaceae*). However, its amide derivative mycousnine enamine is shown to possess the immunosuppressive activity through inhibition of T-cell proliferation but not B-cell proliferation. The amide derivative suppresses the T-cell surface antigen such as the cluster of differentiation (CD69 and CD25) and cytokine such as interleukin-2 and interferon-γ (Wang et al. 2017). The fungal endophyte BAK-I isolated from the bark of plant *Kigelia Africana* (Lam.) showed immunosuppressive activity against TNF-α and cytotoxic activity against Leukaemia-THP-1 and Lung A-549 cancer cell lines (Katoch, Khajuria et al. 2015). In another report, *Penicillium* sp. ZJ-SY associated with the leaf of *Sonneratia apetala* showed immunosuppressive activity, inhibiting the proliferation of B and T lymphocytes, induced by lipopolysaccharide (LPS) and concanavalin A (Con-A), respectively (Liu H et al. 2016).

Immunomodulatory compounds on the other side are the drugs that are used for the treatment of disease by activating the immune response of the body. It is a new source, utilised in the management of immunological disorders. Three bioactive compounds assigned as YS, GS, and BS with immunomodulatory efficiency are reported from fungal endophyte *Pestalotiopsis leucothes*, associated with the host plant *Tripterygium wilfordii*. BS shows immunosuppressive activity by regulating the peripheral blood mononuclear cells (PBMNC) and soluble IL-2 receptor expression that inhibits the production of various cytokines, tumour necrosis factor (TNF)-alpha and interferon (IFN)-gamma (Kumar DSS et al. 2005). In a recent study, four compounds, xanthorrhizol, p-hydroxybenzoic acid, orsellinic acid, and scalarolide, were characterised from the crude extract of fungal endophyte associated with the plant *Ageratum conyzoides*. The crude fungal extract shows antimicrobial activities against *Aspergillus niger, Candida albicans, Salmonella typhi,* and *Pseudomonas aeruginosa* with inhibition zone diameters of 2, 3, 5, 8 mm, respectively. The extract also shows immunomodulatory activity with increase in the level of neutrophils and total white blood cells in mice (Ujam et al. 2019).

### 4.3 Antioxidant compounds derived from fungal endophytes

Bioactive compounds that inhibit oxidation are termed as an antioxidant compound. Bioactive compounds derived from fungal endophytes show excellent property to scavenge the reactive oxygen species (ROS) and/or superoxide radicals. Various fungal endophytes are known to produce polyphenols which are potent inhibitors of oxidation (Khan Abdul Latif et al. 2017). Bioactive compounds with antioxidant activity may also possess antitumor, anti-mutagenic, and anti-inflammatory activities. In one of the study, DPPH scavenging assay revealed that fungal endophytes such as *Diaporthe* sp., *Colletotrichum* sp., and *Arthinium* sp. associated with the plant *Aquilaria subintegra* tend to generate a wide array of bioactive compounds (β-dihydro agarofuran, α-agarofuran, δ-eudesmol, β-agarofuran, and oxo-agarospirol) with strong antioxidant activity (Monggoot et al. 2017). *In vitro* study shows that fungal endophytes of the *Fusarium* genus, associated with the host plant *Fritillaria unibracteata*, have excellent ability to produce antioxidant compounds such as phlorizin, rutin, and gallic acid (Pan et al. 2017). Recently, researchers have shown the apoptotic, cytotoxic, and antioxidant property of ethyl extract of the fungal endophyte *Chaetomium nigricolor* associated with *C. roseus* (Dhayanithy et al. 2019). Thus, fungal endophytes act as natural sources of an antioxidant compounds to play a crucial role in chemoprotection against cancer and other oxidative-damage-associated diseases.

### 4.4 Fungal-endophyte-derived anti-diabetic compounds

Diabetes is a metabolic disease caused by elevated levels of blood sugar (or blood glucose), resulting in severe damage to nerves, eyes, blood vessels, heart, and kidneys. Generally, there are two types of diabetes – Type I and Type-II. More than 95% of diabetes is of type-II diabetes or non-insulin-dependent diabetes (Bailey and Day 1989). The anti-diabetic drugs (miglitol, voglibose, and acarbose) used have shown various side effects *viz*. diarrhoea, abdominal discomfort. The metabolites isolated from the fungal endophytes are seen to exhibit anti-diabetic activity (Lebovitz 1997; Kimura et al. 2012). Fungal endophytes that reside inside the plants are known to produce bioactive metabolites which help in lowering the blood glucose level with antidiabetic nature. Fungal endophyte *Streptomyces* sp. associated with the stem and leaves tissues of *Rauwolfia densiflora* and *Leucas ciliata* plants from Western Ghats of India produces bioactive compound having α-amylase inhibitory property. The extract isolated from *Streptomyces* sp. showed increased uptake of glucose, therefore, acts as an anti-diabetic agent (Akshatha et al. 2014). The fungal endophyte *Nigrospora oryzae* associated with *Combretum dolichopetalum* produces 4-des-hydroxyl altersolanol A, 7-hydroxy-abscisic acid, and 2-cis-4-trans-abscisic acid; all these compounds are known to have anti-diabetic activity (Uzor et al. 2017). Alpha glucosidase inhibitor reduces the absorption of glucose in digestive organ by acting as a carbohydrate hydrolysing enzyme. The fungal endophyte *Xylariaceae* sp. QGS 01 associated with the stem tissue of *Quercus gilva* is known to produce an antidiabetic compound 8-hydroxy-6,7-dimethoxy-3-methylisocoumarine (Indrianingsih and Tachibana 2017). Results of the anti-diabetic assay revealed that the compound has a potential to inhibit α-glucosidase from *Saccharomyces cerevisiae* with an IC_50_ value of 41.75 μg/mL (Indrianingsih and Tachibana 2017). In one of the recent report from our lab, antidiabetic activity of the mycosterol derived from fungal endophyte *Fusarium equiseti* associated with the host plant *Gymnema sylvestre* show competitive inhibition against α-glucosidase and α-amylase (Ranjan et al. 2019).

### 4.5 Bioactive compounds derived from fungal endophytes against cardiovascular diseases

Cardiovascular disease (CVD) is an ever-growing and one of the leading causes of death all around the world. One in four deaths occurs in India due to CVD with 28.1% of the total death and 14.1% of the total disability-adjusted life-years (DALYs) in 2016 as compared with 15.2% of total death and 6.9% of DALYs death in 1990 (Prabhakaran et al. 2018). The use of conventional approaches is proven not to be sufficient for treatment of these diseases, driving us to explore new and effective medicines from fungal endophytes. Statins are a group of drugs that decreases the cholesterol level in the blood. It includes lovastatin and compactin (Barrios-González and Miranda 2010). Lovastatin, a polyketide metabolite, also known as ‘Merck’s Mevacor’, is an anti-cholesterol agent. It is produced from a wide range of fungal endophytes including *Aspergillus flavus, Aspergillus niger, Aspergillus terreus, Trichoderma viride, Monascus ruber, Penicillium* sp., *Monascus* sp, *Monascus ruber, Pleurotuso streatus, Cinnamomum* sp. (Amin-Hanjani et al. 2001). It inhibits the level of rate-limiting enzyme 3-hydroxy-3-methyl-glutaryl-CoA (HMG-CoA) reductase that participates in cholesterol biosynthesis and converts HMG CoA to mevalonate. Lovastatin stops cholesterol synthesis by acting as a competitive inhibitor and is used in the treatment of patients suffering from hypercholesterolaemia (Alberts 1988). Compactin, also called mevastatin isolated from *Penicillium* sp., *Pythium ultimum,* and *Colletotrichum* sp., is an HMG-CoA reductase inhibitor. It lowers serum cholesterol and decreases stroke and cardiovascular disease (Amin-Hanjani et al. 2001). Compactin is not used as a medicine but pravastatin is used as a drug and is commercially available by trade name Zocor (Barrios-González and Miranda 2010). It is produced from endophytic fungus *Aspergillus terreus* and is a lipid-lowering cardiovascular drug. It inhibits the stimulatory activity of angiotensin II on NADPH oxidase and thereby prevents the production of superoxide radicals. It prevents stroke, heart attacks, myocardial infarction, and treats dyslipidemia (Gazzerro et al. 2012).

*In vitro* and *in vivo* study show that ternatin (N-methylated cyclic peptide), isolated from endophytic fungi *Coriolus versicolor*, inhibits the accumulation of fat in an adipose tissue cell line (3T3-L1) and reduces mice fat mass (Ito et al. 2009). Nicotinamide riboside is isolated from the endophytes like *Saccharomyces cerevisiae, Piriformospora* sp., *Epichloe* sp., and *Colletotrichum* sp. of *Bacopa monnieri* and *Azadirachta indica* plants (Chi and Sauve 2013). Administration of nicotinamide riboside in a mice model of cardiomyopathy, that lacks transferrin receptor protein 1 (TfR1) in the heart, resulted in prevention of cardiomegaly, mitochondrial respiration poor cardiac function, and impaired mitophagy (Xu W et al. 2015). The administration of nicotinamide riboside in a mice model of cardiomyopathy, lacking transferrin receptor protein 1 (TfR1) in the heart, has shown to prevent cardiomegaly, mitochondrial respiration, poor cardiac function, and impaired mitophagy (Xu et al. 2015).

### 4.6 Fungal-endophytes-derived bioactive compounds with antimicrobial activity

An antimicrobial agent includes any natural or artificial agent that kills or prevents the growth of other microorganisms. It is grouped on the basis of the microorganism on which it attacks, mode of action, biological activity, and peptide characteristics. Based on biological activity, it is classified in antibacterial, antiviral, antifungal, antitumor, antiparasitic, and insecticidal (Chung and Khanum 2017; Deshmukh et al. 2018). Fungal endophytes are known for the production of bioactive compounds for better substitution of conventional antibiotics. These compounds are known to invade and kill the harmful disease-causing microorganisms that affect humans and animals. *Cryptosporiopsis quercina*, a fungal endophyte associated with *Tripterigeum wilfordii* plants, exhibits antifungal activity against fungal pathogens *Trichophyton* sp. and *C. albicans* because of the production of peptide cryptocandin (Strobel Gary A, Miller R Vincent, et al. 1999a, 1999b). Another group of endophytic fungi, residing in *Quercus variabilis,* produces bioactive compounds that show growth inhibition to pathogenic fungi such as *A. niger, Trichophyton rubrum, Microsporum canis, C. albicans*, and *Epidermophyton floccosum* and bacteria such as *B. subtilis, Escherichia coli,* and *Pseudomonas fluorescens*. Endophytic fungus *Colletotrichum gloeosporioides* associated with the stem of *Artemisia mongolica* is known to produce colletotric acid. Colletotric acid inhibits the growth of *B. subtilis, Sarcina lutea,* and *Staphylococcus aureus* (Zou et al. 2000). Fungal endophyte *Streptomyces* NRRL 3052, associated with *Kennedia nigriscans* (snakevine), produces wide spectrum antibiotics Munumbicins A, B, C, and D. These antibiotics are used against *Mycobacterium tuberculosis, Bacillus anthracis,* and also against malaria parasite *Plasmodium falciparum* (Castillo et al. 2002). Endophytic fungus *Emericella* sp. (HK-ZJ), associated with a mangrove plant *Aegiceras corniculatum*, is known to produce six iso-indoles derivatives along with austinol, dehydroaustin, austin, acetoxydehydroaustin, and aspernidine A and B. This plant shows antiviral activities against influenza A virus (Zhang G et al. 2011). Endophytic metabolite hydroanthraquinone derivative, 6-O-demethyl-4-dehydroxyaltersolanol A, 7-hydroxy-3, didehydrochermesinone B, 7-dimethyl isochromene-6,8-dione obtained from endophytic fungus *Aconitum carmichaelii* associated with *Nigrospora* sp. exhibits inhibitory effect on influenza viral stain and thus used in development of anti-influenza A virus drugs (Zhang S-P et al. 2016). Recently, *Hypericum perforatum* has been screened for the antimicrobial activity of bioactive compounds derived from endophytic fungi. The HPLC-UV analysis revealed that one of the isolates (SZMC 23,769) has the ability to produce hypericin (320.4 ng/mg dry weight mycelia) while three others could synthesise emodin. The fungal endophytes of *Alternaria* sp. have ability to biosynthesise emodin while *Epicoccum nigrum* has ability to synthesise both the bioactive compounds (Vigneshwari et al. 2019). In another study, the bioactive compound produced from endophytic fungi *Alternaria alternata* associated with leaf tissues of the plant *Catharanthus roseus* was explored for anti-microbial and anti-mycotoxigenic activities (Sudharshana et al. 2019). Recently, our lab has isolated and identified active fungal endophytes such as *Colletotrichum* sp., *Cladosporium* sp., and *Fusarium* sp. associated with *Moringa oleifera* while *Alternaria* sp. and *Fusarium* sp. associated with *Withania somnifera*. The ethanolic extract of isolated fungal endophytes *Colletotrichum* sp. of *M. oleifera* and *Alternaria* sp. of *W. somnifera* have shown antibacterial activity against both *E. coli* and *Staphylococcus aureus* (Atri et al. 2020). Different types of endophytic fungi and its action against microorganism are listed (**Table 2**).

**Table 2.** Fungal endophytes associated with host plants and their biological activities against microorganisms

Plants	Endophytic fungi	Active against microorganism	Activity	References
*Solanum mauritianum*	*Paracamarosporium leucadendri, Aureobasidium pullulans, Hyalodendriella* sp.	*Mycobacterium bovis, Mycobacterium smegmatis,* *Candida albicans*	Antibacterial and antimycobacterial	(Pelo et al. 2020)
*Tripterigeum wilfordii*	*Cryptosporiopsis* cf. Quercina	*Trichophyton* sp., *Cryptococcus neoforrnans, Aspergillus fumigatus*	Antifungal	(Strobel et al. 1999b)
*Nyctanthes arbour-tristis*	*Alternaria alternate*, *Nigrospora oryzae*, *Colletotrichum dematium* and *Chaetomium globosum*	*Shigella* sp., *Pseudomonas aeruginosa, Salmonella enteritidis, Salmonella paratyphi*,	Antibacterial and antifungal	(Gond et al. 2012)
*Solanum Nigrum*	*Zygo Rhizopus* sp.	*Pseudomonas* sp., *Staphylococcus aureus, E. coli* and *Pseudomonas aeruginosa*	Antibacterial	(Sunkar and Nachiyar 2011)
*Writhtia tinctoria*	*Aspergillus* sp.	*P. aeruginosa, Pseudomonas fluorescens*	Antibacterial	(Sunkar and Nachiyar 2011)
*Plumbago zeylanico*	*Rhizopus* sp.	*P. aeruginosa, P. fluorescens*	Antibacterial	(Sunkar and Nachiyar 2011)
*Aravae lanata*	*Aspergillus* sp.	*P. aeruginosa, P. fluorescens*	Antibacterial	(Sunkar and Nachiyar 2011)
*Aralia elata*	*Diaporthe, Alternaria*	*Staphylococcus aureus*	Antibacterial	(Wu et al. 2012)
*Laguncularia racemosa*	*Diaporthe phaseolorum*	*S. aureus* and *Salmonella typhi*	Antibacterial	(Sebastianes et al. 2012)
*Panax ginseng*	*Paecilomyces* sp.	*Trichophyton rubrum, A. fumigatus*	Antifungal	(Xu et al. 2009)
*Cinnamomum zeylanicum*	*Muscodor albus*	*Rhizoctonia solani, Pythium ultimum* and *Fusarium oxysporum*	Antifungal	(Strobel et al. 2001)
*Michelia champaca*	*Colletotrichum gloeosporioides*	*Cladosporium cladosporioides* and *C. sphaerospermum*	Antifungal	(Chapla et al. 2014)
*Kandelia candel* (L.) Druce	*Talaromyces* sp.	*Pseudomonas aeruginosa, Sarcina ventriculi, E. coli*	Antibacterial and antifungal	(Liu et al. 2010)
*Calotropis gigantea*	*Alternaria destruens* (AKL-3)	*S. enteric, Sh. flexneri, E. coli, S. aureus*	Antibacterial	(Kaur et al. 2020)
*Ocimum* species (Tulsi)	*–*	*Bacillus cereus, S. aureus, P. aeroginosa, Mycobacterium smegmatis* and *C. albicans*	Antibacterial	(Pavithra et al. 2012)
*Indigofera suffruticosa* Miller	*Nigrospora sphaerica, Pestalotiopsis maculans*	*Staphylococcus aureus*	Antibacterial	(Santos et al. 2015)

## 5. Challenges in the field of fungal endophyte research

Fungal-endophyte-derived bioactive compounds are a source of many novel drugs with low toxicity to combat human diseases. However, approximately 1% of total endophytes have been studied so far, and still millions of fungal endophytes need to be studied and characterised. Production of bioactive compounds from fungal endophytes on industrial scale is a tedious task; therefore, it needs more efficient and advanced approaches like CRISPR-Cas9 and epigenetic modifier for the enhanced production of bioactive compounds (Magotra et al. 2017). Several other strategies such as optimisation of culture parameter, use of elicitors, and co-culture fermentation have also been used to enhance the production of bioactive compounds from fungal endophytes in laboratory conditions. Furthermore, it has always been challenging to isolate and characterise the promising fungal endophytes which have the capacity to produce bioactive compound(s). The application of the molecular approach along with bioinformatics (such as phylogenetic studies) can resolve the problem of identification of fungal strains at species level. The identification of fungal endophytes at the level of species and genus could be assisted by using various DNA barcodes including internal transcribed spacer (ITS) regions, COX2 (partner DNA barcode along with ITS), β-actin, glyceraldehyde 3-phosphate dehydrogenase (house-keeping gene), and region of translational elongation factor 1α (TEF1α) (Schoch et al. 2012; Hoang et al. 2019; Sundaresan et al. 2019). The advantages of approaching DNA barcode based on ITS are the availability of a number of databases, appropriate length of the fragment, and increased amplification of all lineages of fungal endophytes using universal primers (Schoch et al. 2012). However, the use of ITS emerges with certain disadvantages such as a range of intra- and inter-specific distances among the unlike fungal groups, which we need to overcome in the future.

## 6. Conclusion and future perspective

Fungal endophytes associated with plants are considered as an important part of microbial diversity due to their biosynthetic capability of bioactive compounds. The bioactive compounds derived from fungal endophytes can be an essential source of drug formulation or for novel drug discovery. The compounds produced from endophytes play a significant role in human health care such as in cancer, diabetes, disease related to microbes, oxidative stress, and inflammation. In the present era of arising of new diseases, fungal endophytes are an alternative source for the production of natural compounds. The present review highlights that several plants harbour fungal endophytes which synthesise several pharmaceutically important bioactive compounds. The compounds are generally produced in low quantity and in order to produce them in large amount several biotechnological tools such as TALEN (Transcription Activator-Like Effector Nucleases), CRISPR (Clustered Regulatory Interspaced Short Palindromic Repeats)-Cas9, and ZFN (Zinc-finger nucleases) are used. Genetic manipulations could also be possible through adopting other techniques such as *in vitro* regeneration, electroporation technology for the production of transgenic medicinal plants, combinatorial biosynthesis, and genetic transformations. In future, studies should be focused on the mechanism behind the plant–endophyte interaction, biogeographical pattern of endophytes, revealing the basic mechanism of synthesis of bioactive compounds and also the strategies to manipulate the identified pathways for the derivation of natural bioactive compounds from fungal endophytes.
